# Bedrock geology affects foliar nutrient status but has minor influence on leaf carbon isotope discrimination across altitudinal gradients

**DOI:** 10.1371/journal.pone.0202810

**Published:** 2018-09-19

**Authors:** Renato Gerdol, Paola Iacumin, Rita Tonin

**Affiliations:** 1 Department of Life Sciences and Biotechnology, University of Ferrara, Ferrara, Italy; 2 Department of Chemistry, Life Sciences and Environmental Sustainability, University of Parma, Parma, Italy; 3 Faculty of Science and Technology, Free University of Bolzano, Bozen-Bolzano, Italy; University of Alberta, CANADA

## Abstract

Carbon isotope discrimination (Δ^13^C) in plant leaves generally decreases with increasing altitude in mountains. Lower foliar Δ^13^C at high elevation usually is associated with higher leaf mass per area (LMA) in thicker leaves. However, it is unclear if lower foliar Δ^13^C in high-altitude plants is caused by improved photosynthetic capacity as an effect of higher nutrient, especially nitrogen, content in thicker leaves. We investigated trends of foliar Δ^13^C in four species, each belonging to a different plant functional type (PFT), across two altitudinal gradients, each on a different bedrock type (carbonate and silicate bedrock, respectively) in a region of the southern Alps (Italy) where the foliar Δ^13^C was not affected by water limitation. Our objective was to assess whether the altitudinal patterns of foliar Δ^13^C in relation to leaf morphology and foliar nutrients were conditioned by indirect control of bedrock geology on soil nutrient availability. The foliar Δ^13^C of the four species was mainly affected by LMA and, secondarily, by stomatal density (SD) but the relative importance of these foliar traits varied among species. Area-based nutrient contents had overall minor importance in controlling C discrimination. Relationships among foliar Δ^13^C, foliar nutrient content and leaf growth rate strongly depended on soil nutrient availability varying differently across the two gradients. In the absence of water limitation, the foliar Δ^13^C was primarily controlled by irradiance which can shape anatomical leaf traits, especially LMA and/or SD, whose relative importance in determining C isotope discrimination differed among species and/or PFT. Decreasing foliar Δ^13^C across altitudinal gradients need not be determined by improved photosynthetic capacity deriving from higher nutrient content in thicker leaves.

## Introduction

Carbon (C) isotope discrimination (Δ^13^C) in C_3_ plants reflects the ratio between internal CO_2_ partial pressure in the leaves (p_i_) and external CO_2_ partial pressure in the atmosphere (p_a_). Leaves of plants operating at lower p_i_/p_a_ ratio discriminate less the heavy C isotope (^13^C) and thus present lower Δ ^13^C signature. The foliar Δ^13^C of terrestrial plants generally decreases with increasing altitude in mountains [1, 2]. However, there still is uncertainty about the causes accounting for variations in foliar Δ^13^C in relation to altitude. Lower p_i_/p_a_ ratio, and correspondingly lower foliar Δ^13^C, can be achieved by three different mechanisms: (i) reduced stomatal conductance (g_s_), (ii) reduced mesophyll conductance (g_m_) and (iii) increased carboxylation efficiency [3]. Several features related to leaf morphology, physiology and foliar nutrient status, often interacting with one another, can affect the foliar Δ^13^C across altitudinal gradients. Stomatal density (SD) and stomatal length (SL) often increase at high altitude in order to enhance the photosynthetic performance (see [4, 5] but see also [6]). In particular, increasing SD can also represent an adaptation to improve the interception of solar radiation with increasing altitude [7]. Whatever the cause, higher SD and/or SL can enhance C isotope discrimination. Leaf mass per area (LMA) in plants from high altitudes generally is greater than in plants from low altitudes in association with thicker mesophyll cell walls, higher mesophyll cell density and consequently higher C content per unit leaf area (C_area_). This hampers CO_2_ transfer in the leaves, thus resulting in lower p_i_/p_a_ through reduced mesophyll conductance (g_m_) [8, 9, 10]. Negative correlation between foliar Δ^13^C and LMA can be further explained considering relationships of LMA with other environmental factors besides temperature. The LMA increases under high light levels because more photosynthetic biomass per unit leaf area improves C gain under high irradiance. The LMA also increases with reduced water availability as an effect of smaller transpiring surface and more tightly packed tissues, which represents an effective adaptation to drought stress in water-limited ecosystems [11].

Lowering of foliar Δ ^13^C can also be achieved by higher growth potential if carboxylation efficiency increases at high altitudes [12]. Relationships between carboxylation efficiency and altitude can be in turn mediated by leaf morphology. Increasing LMA with increasing altitude often implies parallel increase in the amount of N per unit leaf area (N_area_) and in carboxylation efficiency [12]. Other studies, although supporting the general trend of increasing LMA at high altitude, did not observe a parallel increase in carboxylation efficiency with the latter not varying [8], or even decreasing [10, 13] across altitudinal gradients. These findings suggest that, while high-LMA in thick leaves is always associated with high amount of support structures, the fraction of N allocated to functional cell compartments can vary depending on soil nutrient status [14]. Relationships between LMA and carboxylation efficiency may also be affected by the availability of other nutrients. While a number of studies have investigated altitudinal patterns of foliar Δ ^13^C in relation to environmental factors that vary in relation to altitude, very few of them have considered bedrock geology as a possible driver of variation in foliar Δ ^13^C across altitudinal gradients. Indeed, bedrock geology plays a major role in determining the supply of rock-derived nutrients such as calcium, magnesium, potassium and especially phosphorus (P) [15]. In a recent paper we observed that bedrock geology strongly affected foliar P concentrations, but not foliar N concentrations, across altitudinal gradients on two different parent materials. In particular, foliar P concentrations strongly increased with increasing altitude on carbonate bedrock and slightly decreased with increasing altitude on silicate bedrock. This strongly conditioned the growth potential of plant species that showed an increasing trend across the altitudinal gradients on silicate bedrock and a decreasing trend across the altitudinal gradient on carbonate bedrock [16].

**Fig 1:**
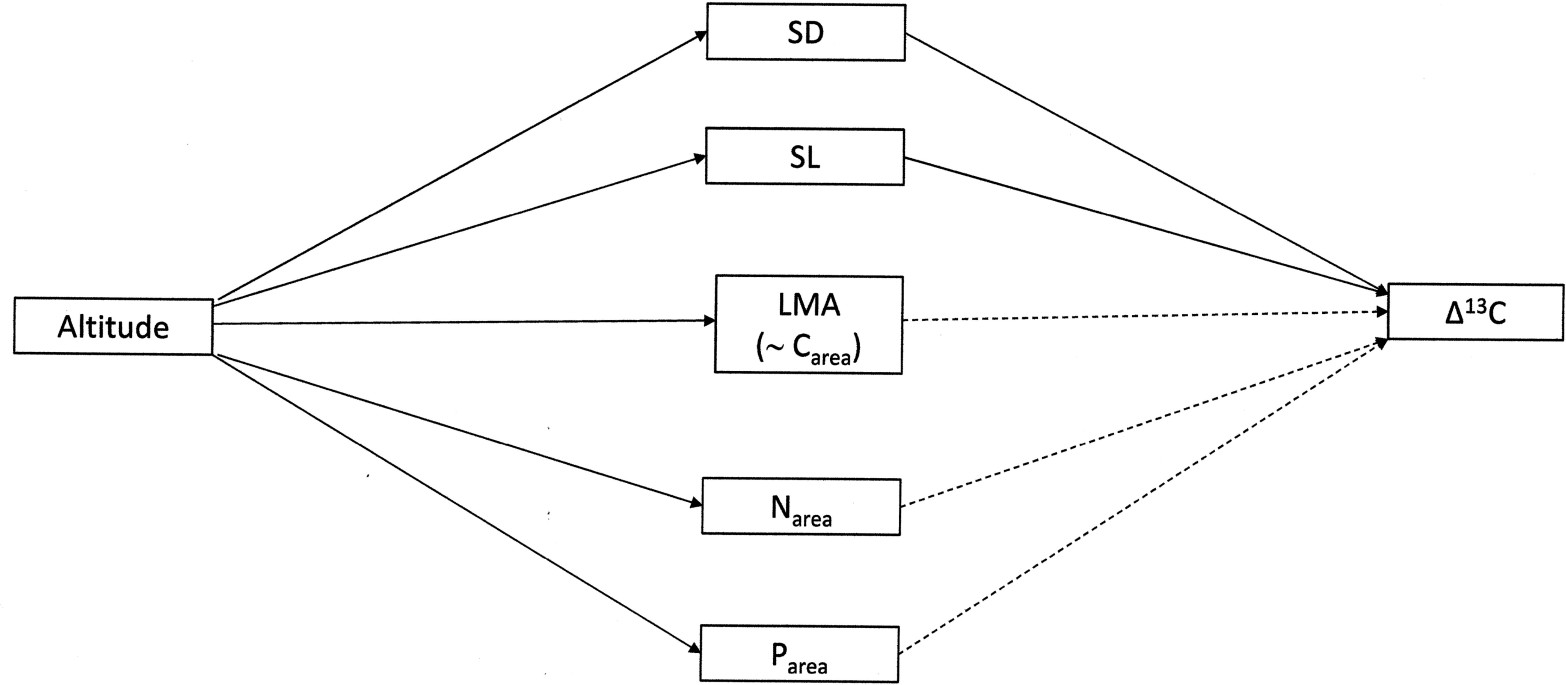
Expected effects of increasing altitude on foliar Δ^13^C mediated by changes in leaf morphology and foliar nutrient status. Full arrows indicate positive effects, dashed arrows indicate negative effects.

The general purpose of this study was to investigate altitudinal trends of foliar Δ^13^C in relation to leaf features in four species, each belonging to a different plant functional type (PFT). We hypothesized that the foliar Δ^13^C presented positive relations with SD and SL, and negative relations with LMA and area-based nutrient concentrations (C_area_, N_area_ and P_area_; Fig 1). We also hypothesized that, if bedrock geology affects the foliar Δ^13^C by influencing the growth potential through an indirect effect on foliar nutrient status, this will imply significant correlations between foliar Δ^13^C and leaf growth rates with the sign of the correlations varying in relation to soil nutrient availability. As the rate of change in foliar Δ^13^C across altitudinal gradients may vary among both plant functional types (PFT) and species within a given PFT [2], an additional objective of our study was to assess whether the altitudinal trends of foliar Δ^13^C were consistent among species and/or PFTs. A list of the abbreviations used throughout the paper is given in Table 1.

**Table 1:** List of the abbreviations for variables used in text, tables and figures.

Abbreviation	Variable	Unit
***Measured variables***		
**Δ**^**13**^**C**	Carbon (C) isotope discrimination	‰
**SD**	Stomatal density	mm^-2^
**SL**	Stomatal length	μm
**LMA**	Leaf mass per area	g m^-2^
**C**_**area**_	C content per unit leaf area	mg cm^-2^
**N**_**area**_	Nitrogen (N) content per unit leaf area	μg cm^-2^
**P**_**area**_	Phosphorus (P) content per unit leaf area	μg cm^-2^
**Soil T**	Soil temperature	°C
**Soil VWC**	Soil volumetric water content	%
**PPFD**	Photosynthetic photon flux density	μmol m^-2^ s^-1^
***Not-measured variables***		
**p**_**i**_	Internal CO_2_ partial pressure	Pa
**p**_**a**_	External CO_2_ partial pressure	Pa
**g**_**s**_	Stomatal conductance	mmol m^-2^ s^-1^
**g**_**m**_	Mesophyll conductance	mmol m^-2^ s^-1^
***Other abbreviations***		
**GRM**	Step-wise multiple regression	-
**PCA**	Principal component analysis	-
**PFT**	Plant functional type	-
**TDR**	Time domain reflectometer	-

## Materials and methods

### Study area and sampling

The study area and the sampling design were thoroughly described in [16]. Briefly, the field study was carried out across two 1000-m wide altitudinal gradients (*ca*. 1200–2200 m above sea level) in the Dolomites (Eastern Alps, Northern Italy; 46°13–25’N, 11°27–43’E). The two gradients were located each on a different bedrock type: carbonate (dolomite and limestone) and silicate (rhyolite and dacite) bedrock, respectively. The climate is cool-montane with mean annual temperature of about 8°C at 1000 m and about 3°C at 2000 m. Mean annual precipitation is 900–1000 mm, mostly concentrated during the summer months when aridity never occurs at any altitude.

As our objective was to compare altitudinal patterns of foliar Δ^13^C among species and PFTs we made a number of preliminary surveys directed to select species occurring with high frequency throughout the gradients on both parent materials. Four species, each belonging to a different PFT, were deemed suitable for our study: *Vaccinium myrtillus* L. (henceforth *V*. *myrtillus*), a deciduous dwarf shrub; *Vaccinium vitis-idaea* L. (henceforth *V*. *vitis-idaea*), an evergreen shrub; *Homogyne alpina* (L.) Cass. (henceforth *H*. *alpina*), a wintergreen forb; *Calamagrostis villosa* (Chaix) Gmelin (henceforth *C*. *villosa*), a grass. Sampling was carried out at six 1-ha sites, located at about 200-m altitude intervals in each of the two transects, with 12 sampling sites in total. The sites from 1200 to 1800 m were located in closed spruce (*Picea excelsa*) forests. The sites at 2000 m were located in more open spruce forests, close to the treeline. The sites at 2200 m were located above the treeline in subalpine scrubs. Five 5 × 5 m plots were set up at each of the 12 sites. The plots were located at least 60 m apart. Ten leaves of all four species were picked in all of the 60 plots, each from a different ramet (for shrubs) or from a different individual (for herbs), on clear sunny days at the end of the growing season (1–2 August 2011). Only fully-developed leaves with no visible sign of damage were sampled. Current-year leaves were sampled for the evergreen and the wintergreen species. Daily leaf growth rates were calculated based on the time elapsed between growth start and sampling (see [16] for details). A soil sample was collected in each of the 60 plots as described in [16]. Permissions for the field study were provided by local authorities (Comune di Pozza di Fassa and Magnifica Comunità di Fiemme).

Soil temperature (T) was recorded continuously during the growing season (May-June 2011) by data loggers (Hobo, Onset Computer Corporation, Bourne, MA, USA) placed at 5-cm depth at each of the 12 sites. Soil volumetric water content (VWC) was measured at each site on an overcast day without precipitation, *ca*. 40 h after a strong rainy event, using a FieldScout time domain reflectometer TDR 100 Soil Moisture Meter (Spectrum Technologies Inc., Aurora, IL, USA). Photosynthetic photon flux density (PPFD) was measured at all sites, from 10 a.m. to 4 p.m. during a clean-sky day, using a radiometer (DO 9721, Delta OHM, Padova, Italy).

### Measurements and chemical analyses

The leaf samples were stored in a refrigerator at 4°C for 12–36 hr. Afterwards, five of the leaves in each sample were scanned using a scanner (HP Scanjet 2400). Leaf area was subsequently measured on the scanned image using the Scion Image software for Windows version Alpha 4.0.3.2 (Scion Corporation, MD, USA). The scanned leaves were oven-dried at 40°C for 48 hr and weighed individually for determining leaf mass and then calculating LMA. The remainder of the five leaves used for microscopy was also oven-dried at 40°C for 48 hr and finally bulked with the material used for determining LMA. A sub-sample of the additional leaves collected at each plot was oven-dried at 105°C and weighed to determine the mass loss between 40°C and 105°C. All mass values are referred to the 105°C weight. The bulked oven-dried samples were powdered, digested in 3 mL of selenous H_2_SO_4_ at 420°C and analysed for total N concentrations by the salicylate method and total P concentration by the molybdenum blue method using a continuous flow autoanalyser (FlowSys; Systea, Anagni, Italy).

The soil samples were sequentially extracted for determining concentrations of different N and P fractions. The analytic procedure and the results are thoroughly presented in Gerdol et al. [16]. Only the data of total soil N and total soil P, extracted and analyzed as for the leaves, are summarized in this study.

Carbon isotope discrimination was calculated according to [17]:

$$\Delta^{13}C=[10^{3}\times (\delta^{13}C_{air}-\delta^{13}C_{sample})÷(1+\delta^{13}C_{sample})]\;‰$$

δ^13^C_air_, measured in the field at a nearby area at 1800 m (Brancaleoni and Gerdol unpublished), was 8.2 ± 0.1 ‰; δ^13^C_sample,_ together with C concentration, was measured in leaves with an elemental analyzer (EA 1110, Carlo Erba, Milan, Italy) coupled online with an isotope ratio mass spectrometer (delta Plus XP, ThermoFinnigan, Bremen, Germany) and expressed as:

$$\delta^{13}C_{sample}={(}^{13/12}R_{sample}÷{}^{13/12}R_{V-PDB})-1$$

where V-PDB is the primary international standard *Belemnitella americana* from the Pee Dee Formation.

Five additional leaves of the four species were used for stomatal measurements. Stomatal measurements were carried out by the clear nail polish method [18]. Preliminary observations showed that in all four species most of the stomata were located in the abaxial side of the leaves, Therefore, only the abaxial side was sampled for stomatal measurements. Two epidermal impressions were taken from the abaxial side of a fully developed leaf, avoiding leaf veins. After removing the nail varnish with transparent adhesive, the epidermal impression was put onto a microscope slide. All measurements were performed using a light microscope (Leica DMLS, Leica Biosystems, Nussloch, Germany), and an attached digital camera (DeltaPix, Måløv, Denmark), at 400 × magnification. SL, i.e. guard cell length, of all stomata was measured using the software DeltaPix, v.3.2.x. To estimate SD, the number of stomata was counted in five different fields of view for each epidermal impression. Thus, SD was calculated on 10 fields of view per leaf according to [19]:

$$SD\;\left(mm^{‑2}\right)=\left(stomatal\;number\;per\;visual\;field\right)/\left(visual\;field\;area\right)$$

### Statistics

The data on soil chemistry, foliar Δ^13^C and foliar traits were analyzed by two-way factorial ANOVAs with altitude, bedrock and their interaction as fixed factors. All data were log-transformed when not meeting the assumption of variance normality as assessed by the Kolmogorov-Smirnov test. Significance of differences among means were assessed using Bonferroni post-hoc tests.

A comparative analysis of mutual relationships among foliar Δ^13^C and foliar traits in the four species across altitudinal gradients was carried out by principal component analysis (PCA). The PCA was done out using the mean values (N = 5) of foliar Δ^13^C and foliar traits in the four species at the 12 sampling sites as computation variables. The four species differed significantly from each other in terms of most variables because of intrinsic differences in foliar traits. Therefore, all variables were normalized prior to the PCA as follows:

$$X_{ni}=\left(X_{oi}÷X_{max}\right)\times 100$$

X_ni_ is the normalized value of the X-variable at the i-site; X_oi_ is the original value of the X-variable at the i-site; X_max_ is the maximum value of the X-variable across the 12 sites.

The PCA was based on the correlation matrix among the normalized variables which provided centred standardized scores for variables and sites.

Step-wise multiple regressions (GRM) of foliar Δ^13^C on foliar traits were run separately for each species. The p-values and F-values for insertion and removal of the explanatory variables were set at 0.05 and 1.0, respectively. The delta-sweep was set at 1.0 × e^-10^. All statistical computations were carried out using the package STATISTICA (Release 6; StatSoft Inc., Tulsa, OK, USA).

## Results

### Altitudinal gradients and environment

Soil T (Table 2) varied linearly in relation to altitude, with an overall correlation close to -1 (r = - 0.99; N = 12; p < 0.001). The PPFD was rather low at all sites in closed forests (*ca*. 1200–1800 m). PPFD was higher at 2000 m and very high at the open high-altitude (*ca*. 2200 m) sites on both parent materials (Table 2). Soil VWC (Table 2) was 20–25% at all sites with no relationships with altitude.

**Table 2:** Environmental variables at the sampling sites.

Bedrock	Altitude (m)	Soil T (°C)	Soil VWC (%)	PPFD (μmol m^-2^ s^-1^)
**CARBONATE**	1200	9.34	23.4	141
	1400	8.88	22.1	154
	1600	7.05	22.3	128
	1800	6.55	21.1	120
	2000	5.29	22.5	860
	2200	4.95	25.5	1895
**SILICATE**	1200	8.48	22.5	174
	1400	7.78	22.6	129
	1600	7.37	22.9	141
	1800	7.09	24.1	112
	2000	6.03	22.1	416
	2200	5.26	21.4	1741

The 12 sampling sites were located across two altitudinal gradients on two different bedrocks (six on carbonate bedrock and six and on silicate bedrock).

Soil temperature (T) was recorded continuously at all sites by data loggers. Soil volumetric water content (VWC) and photosynthetic photon flux density (PPFD) were measured in the field on a clean-sky day and an overcast day without precipitation, respectively.

**Fig 2:**
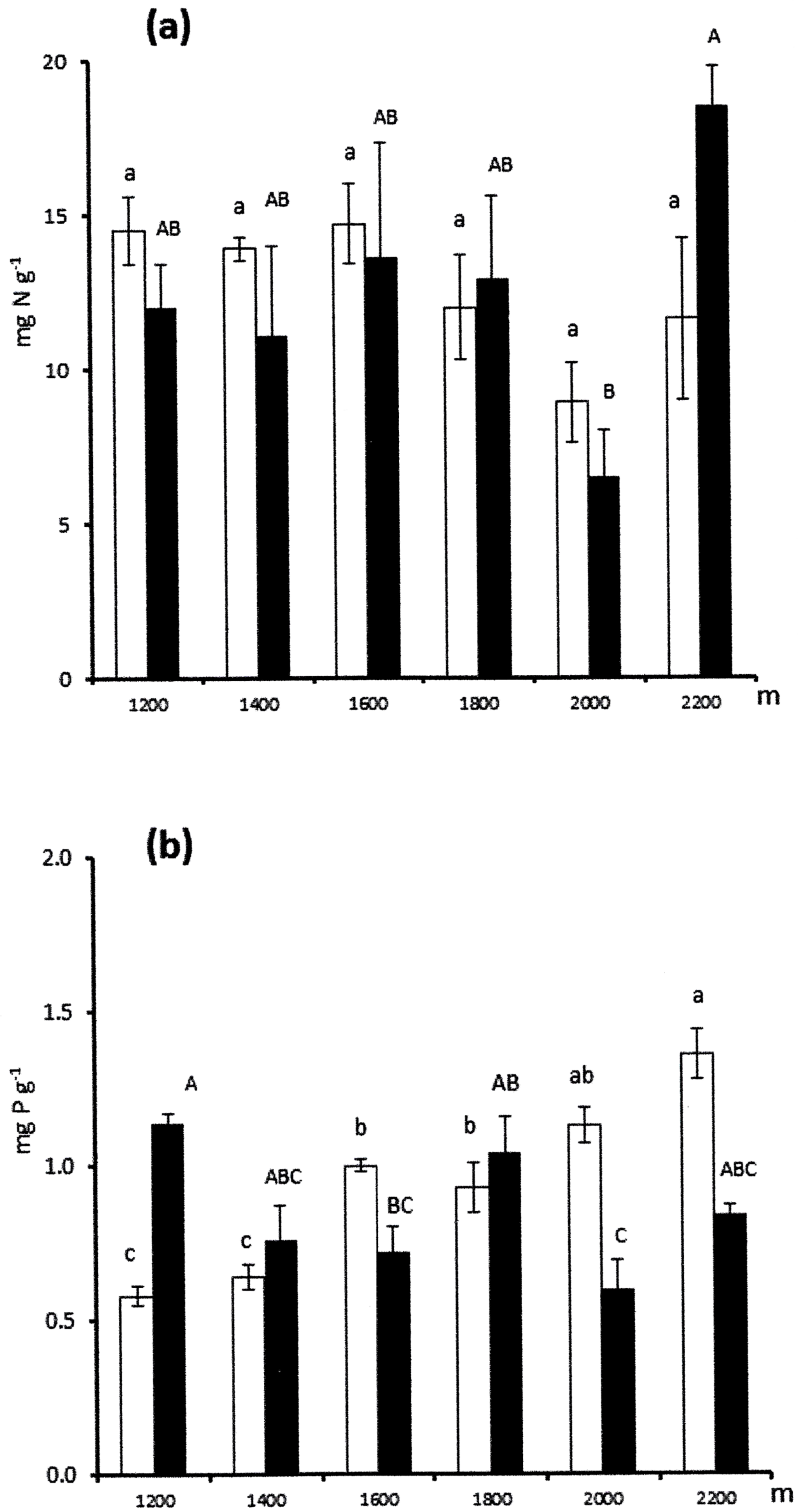
Soil nutrient contents across altitudinal gradients. Mean (± SE) total soil N concentrations (a) and total soil P concentrations (b) across altitudinal gradients on carbonate bedrock (empty bars) and silicate bedrock (full bars). Within each series the means followed by different letters (small letters for carbonate bedrock; capital letters for silicate bedrock) differ significantly (p < 0.05) from each other based on Bonferroni post-hoc tests.

Total soil N concentrations (Fig 2A) differed significantly with altitude (F_5,48_ = 3.16; p = 0.02) but were unaffected by bedrock (F_1,48_ = 0.02; p = 0.90), with no altitude × bedrock interaction (F_5,48_ = 1.69; p = 0.15). Total soil N concentrations were similar on the two parent materials with overall lower values at 2000 m (Fig 2A). Total soil P concentrations (Fig 1B) differed strongly with altitude (F_5,48_ = 6.75; p < 0.001) and were overall higher on carbonate bedrock (F_1,48_ = 4.42; p = 0.04). There were opposite trends of total soil P concentrations across the gradients on the two parent materials, as shown by the significant altitude × bedrock interaction (F_5,48_ = 16.99; p < 0.001). Indeed, total soil P concentrations increased regularly with increasing altitude on carbonate bedrock and fluctuated more erratically across the gradient, with highest value at 1200 m, on silicate bedrock (Fig 2B).

### Foliar Δ^13^C and foliar traits across the gradients

**Fig 3:**
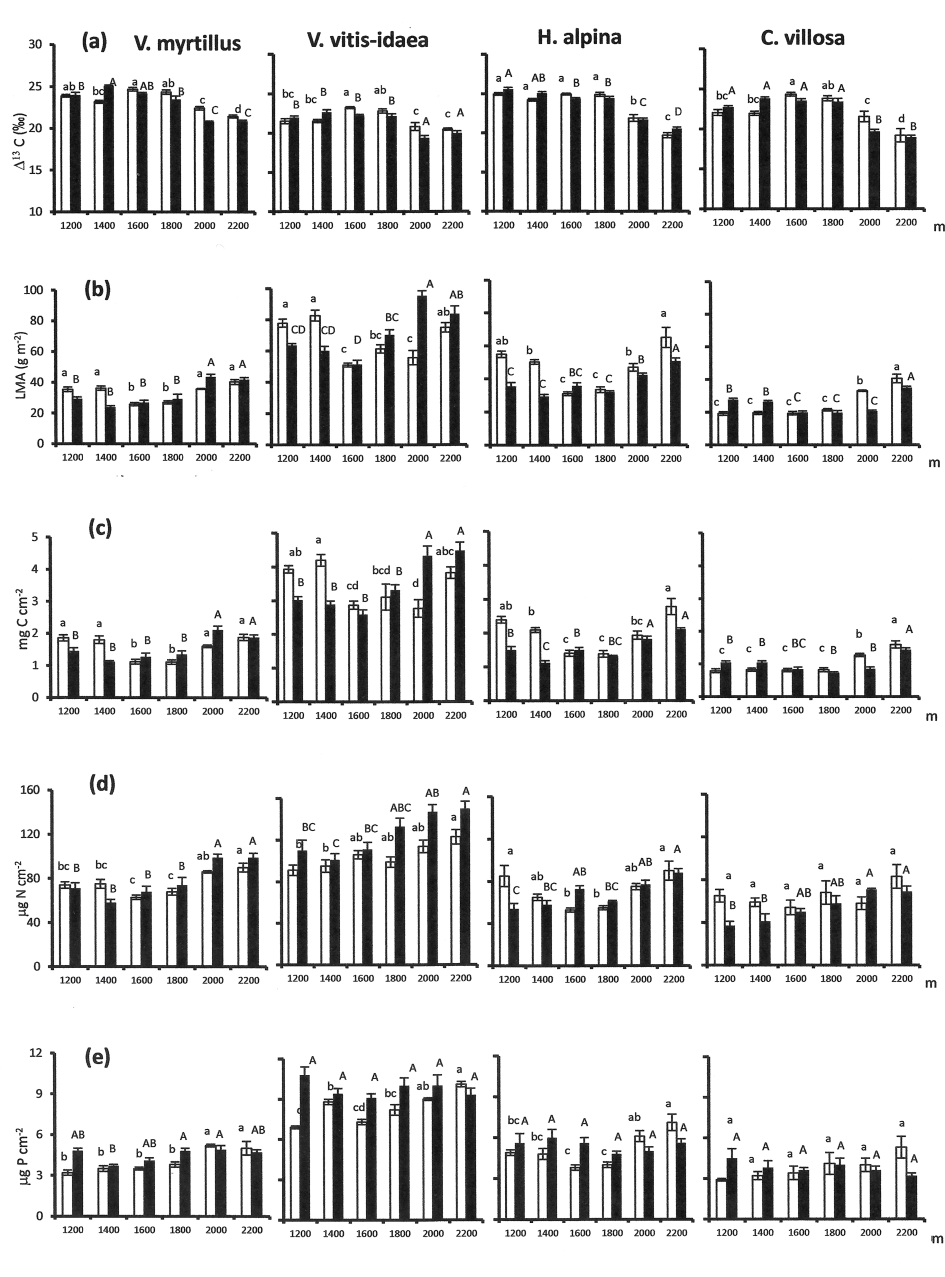
Foliar C isotope discrimination and foliar traits across altitudinal gradients. Mean (± SE) C isotope discrimination (Δ^13^C, a) leaf mass per area (LMA, b) area-based foliar carbon content (C_area_, c), area-based foliar nitrogen content (N_area_, d) and area-based foliar phosphorus content (P_area_, e) across altitudinal gradients on carbonate bedrock (empty bars) and silicate bedrock (full bars) in four species. Within each series the means followed by different letters (small letters for carbonate bedrock; capital letters for silicate bedrock) differ significantly (p < 0.05) from each other based on Bonferroni post-hoc tests.

The foliar Δ^13^C decreased significantly with altitude, and was unaffected by bedrock in all four species (Table 3; Fig 3A). The foliar Δ^13^C of all species was much similar in the 1200–1800 m range, but with somewhat higher values at 1600–1800 m on carbonate bedrock which resulted in a significant altitude bedrock interaction (Table 3). The foliar Δ^13^C was markedly lower at 2000–2200 m in all species (Fig 3A). SD increased consistently with increasing altitude on both bedrock types in *V*. *myrtillus*, *V*. *vitis-idaea* and *H*. *alpina* but did not change significantly across the altitudinal gradients in *C*. *villosa* (Tables 3 and 4). SL varied significantly with altitude only in *V*. *vitis-idaea* (Tables 3 and 4), that showed decreasing SL with increasing altitude on carbonate bedrock (Table 4).

**Table 3:** ANOVA table for C isotope discrimination and foliar traits in the four species.

	n.d.f.	d.d.f.		*V*. *myrtillus*	*V*. *vitis-idaea*	*H*. *alpina*	*C*. *villosa*
**10**^**3**^ **× Δ**^**13**^**C**	5	48	A	91.5**	32.62**	154.2**	45.65**
	1	48	B	2.0	3.44	1.5	0.30
	5	48	A × B	15.4**	5.63**	3.9**	5.22**
**SD**	5	48	A	5.65*	6.24**	9.87**	7.33
	1	48	B	3.16	0.80	0.03	0.24
	5	48	A × B	2.30	0.29	0.08	4.27**
**SL**	5	48	A	1.73	13.94**	1.08	25.49**
	1	48	B	0.03	1.40	0.40	0.83
	5	48	A × B	2.27	5.30**	0.73	8.24**
**LMA**	5	48	A	33.46**	16.84**	31.67**	66.94**
	1	48	B	1.25	1.57	47.35**	0.96
	5	48	A × B	11.52**	17.92**	10.00**	22.97**
**C**_**area**_	5	48	A	20.51**	9.90**	28.67**	40.77**
	1	48	B	0.4	0.02	50.72**	0.61
	5	48	A × B	10.95**	12.55**	9.10**	9.34**
**N**_**area**_	5	48	A	21.89**	10.48**	10.33**	4.49**
	1	48	B	1.20	26.68**	0.40	6.67*
	5	48	A × B	3.72*	1.79	5.77**	2.04
**P**_**area**_	5	48	A	12.59**	2.83*	4.91**	0.74
	1	48	B	11.72**	29.60**	1.69	0.00
	5	48	A × B	4.80**	6.34**	4.59**	2.78*

ANOVA table for C isotope discrimination (10^3^ × Δ^13^C), stomatal density (SD), stomatal length (SL), leaf mass per area (LMA), C content per unit leaf area (C_area_), N content per unit leaf area (N_area_) and P content per unit leaf area (P_area_) in leaves of four species across two altitudinal gradients on two bedrocks. Columns include the numerator degrees of freedom (n.d.f.), the denominator degrees of freedom (d.d.f.) and the F-values for altitude (A), bedrock (B) and their interaction.

Significant values in bold.

* p < 0.05

** p < 0.01

**Table 4:** Stomatal density and stomatal size in leaves of the four species.

		*V*. *myrtillus*		*V*. *vitis-idaea*		*H*. *alpina*		*C*. *villosa*	
		C	S	C	S	C	S	C	S
**SD (mm**^**-2**^**)**	1200 m	86 ± 5 bc	92 ± 10 ab	208 ± 13 b	239 ± 32 b	115 ± 6 b	107 ± 6 c	170 ± 21 a	99 ± 11 a
	1400 m	88 ± 8 bc	79 ± 10 b	254 ± 14 ab	250 ± 25 ab	137 ± 14 ab	130 ± 9 bc	92 ± 6 b	91 ± 23 a
	1600 m	83 ± 6 bc	90 ± 8 ab	264 ± 16 ab	261 ± 14 ab	111 ± 17 b	115 ± 15 bc	67 ± 5 b	77 ± 9 a
	1800 m	71 ± 3 c	111 ± 16 ab	255 ± 8 ab	270 ± 16 ab	129 ± 7 ab	126 ± 11 bc	65 ± 6 b	81 ± 7 a
	2000 m	109 ± 8 ab	127 ± 8 a	312 ± 26 a	335 ± 11 a	168 ± 20 ab	172 ± 17 ab	91 ± 8 b	90 ± 6 a
	2200 m	115 ± 6 a	105 ± 5 ab	290 ± 24 ab	288 ± 18 ab	189 ± 21 a	191 ± 17 a	69 ± 7 b	97 ± 6 a
**SL (μm)**	1200 m	16.9 ± 0.5 A	19.1 ± 0.7 A	16.7 ± 0.3 A	14.9 ± 0.5 A	18.2 ± 0.9 A	18.6 ± 0.6 A	19.0 ± 2.4 B	30.1 ± 1.3 BC
	1400 m	19.5 ± 0.5 A	18.8 ± 0.4 A	14.9 ± 0.3 B	13.7 ± 0.4 A	18.2 ± 0.6 A	17.7 ± 0.6 A	37.8 ± 0.8 A	27.3 ± 1.5 C
	1600 m	19.4 ± 0.6 A	18.9 ± 0.3 A	13.5 ± 0.4 C	14.0 ± 0.3 A	20.0 ± 1.1 A	18.5 ± 0.4 A	37.6 ± 2.8 A	36.5 ± 2.0 AB
	1800 m	19.2 ± 1.0 A	19.0 ± 0.6 A	13.4 ± 0.2 C	13.6 ± 0.3 A	18.6 ± 0.9 A	17.8 ± 0.6 A	41.0 ± 1.4 A	42.3 ± 2.0 A
	2000 m	19.4 ± 0.7 A	19.5 ± 0.5 A	12.6 ± 0.3 C	13.8 ± 0.2 A	18.6 ± 0.4 A	19.4 ± 0.7 ±	38.3 ± 1.3 A	35.6 ± 1.0 AB
	2200 m	19.4 ± 0.3 A	18.3 ± 0.3 A	13.8 ± 0.3 BC	13.6 ± 0.4 A	18.8 ± 0.4 A	19.1 ± 0.8 A	42.0 ± 1.4 A	38.5 ± 1.9 A

Mean (± SE) stomatal density (SD) and stomatal length (SL) measured in the abaxial side of five leaves at five plots in twelve sampling sites across two altitudinal gradients, six on carbonate bedrock (C) and six and on silicate bedrock (S).

Within each column, for each of the two variables (SL capital letters, SD small letters) the means followed by different letters differ significantly (p < 0.05) from each other based on Bonferroni post-hoc tests.

LMA, C_area_, and to a certain extent N_area_ as well, mirrored the altitudinal trends of foliar Δ^13^C with higher values at the sites where foliar Δ^13^C was lower (Table 3; Fig 3B–3D). Conversely, P_area_ varied differently across the gradients on the two bedrock types with further differences among species (Table 3; Fig 3E). P_area_ was overall higher on silicate bedrock in *V*. *myrtillus* and *V*. *vitis-idaea* but not in *H*. *alpina* and *C*. *villosa*. P_area_ did not vary across the altitudinal gradient on silicate bedrock in any species but increased with increasing altitude on carbonate bedrock in all species except *C*. *villosa* (Fig 3E).

### Relationships among foliar Δ^13^C and foliar traits

**Fig 4:**
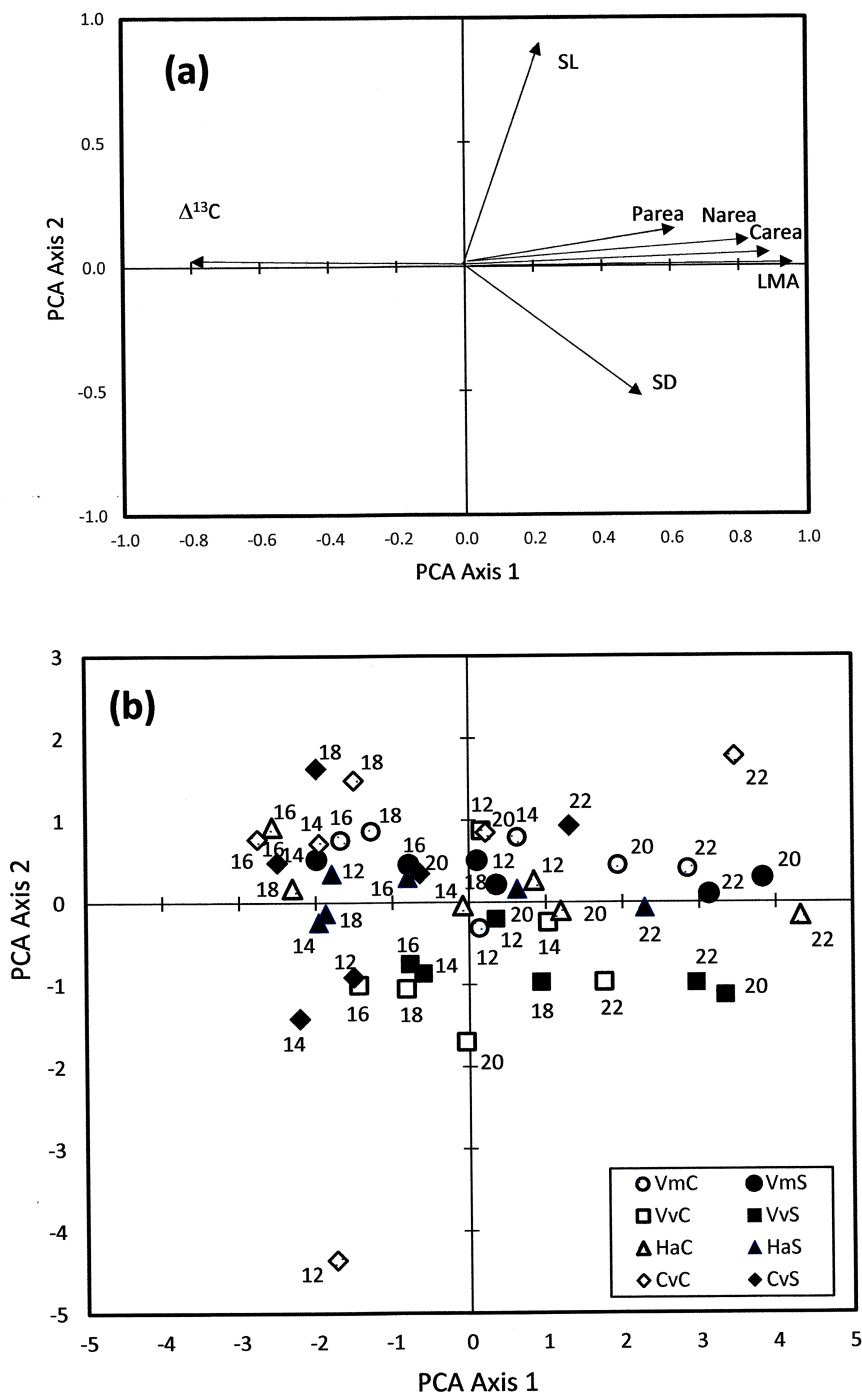
Multivariate statistical analysis of mutual relationships among C isotope discrimination and foliar traits across altitudinal gradients. Principal component analysis (PCA) of normalized mean values of C isotope discrimination (Δ^13^C) leaf mass per area (LMA) area-based foliar carbon content (C_area_), area-based foliar nitrogen content (N_area_), area-based foliar phosphorus content (P_area_, e), stomatal length (SL) and stomatal density (SD) across altitudinal gradients on carbonate bedrock and silicate bedrock. Variable scores (a) and site scores (b) at six altitudes (12: 1200 m; 14: 1400 m; 16: 1600 m; 18: 1800 m; 20: 2000 m; 22: 2200 m) on two bedrock types (empty symbols: carbonate bedrock; full symbols: silicate bedrock) in four species (Vm: *Vaccinium myrtillus*; Vv: *Vaccinium vitis-idaea*; Ha: *Homogyne alpina*; Cv: *Calamagrostis villosa*). The first PCA axis accounts for 52.4% of total variance. The second PCA axis accounts for 14.8% of total variance.

The PCA revealed close relationships between foliar Δ^13^C on one side, and LMA, C_area_ and N_area_ on the other side (Fig 4A). Indeed, the foliar Δ^13^C presented strong negative correlation with the first PCA axis whereas LMA, C_area_ and N_area_ all presented strong positive correlations with the first PCA axis. Such strong correlations of the four variables with the first PCA axis were documented by long vectors directed almost parallel to the axis (Fig 4A). P_area_ also was positively correlated with the first PCA axis, but the correlation strength was weaker as shown by the shorter P_area_ vector slightly diverging from the first PCA axis (Fig 4A). SD and SL were more closely related to the second PCA axis, with SL showing positive correlation and SD negative correlation with the second PCA axis, respectively. The uppermost sites (2200 m and, to a lesser extent, 2000 m) had high scores on the first PCA axis and were thus located at the right end of the PCA diagram (Fig 4B). Conversely, the sites in the 1200–1800 m range had lower scores on the first PCA axis and were scattered with no clear pattern in the left part of the PCA diagram (Fig 4B).

The F-values of the stepwise multiple regressions revealed strongly significant effects of LMA on foliar Δ^13^C in all species (Table 5). SD did exert a significant effect on the foliar Δ^13^C of *V*. *vitis-idaea* and especially *H*. *alpina* but not on the foliar Δ^13^C of *V*. *myrtillus* and *C*. *villosa* (Table 5) SL did not influence foliar Δ^13^C in any species (Table 5). C_area_, N_area_ and P_area_ all had minor influence on foliar Δ^13^C (Table 5) because of their high multi-collinearity with LMA (Fig 3A).

**Table 5:** GRM table for multiple regressions of C isotope discrimination on leaf mass per area and foliar nutrient contents in the four species.

	d.f.	*V*. *myrtillus*	*V*. *vitis-idaea*	*H*. *alpina*	*C*. *villosa*
**LMA**	1	14.73**	26.89**	16.93**	33.81**
**SD**	1	0.87	6.45**	38.06**	3.68
**SL**	1	1.87	2.04	1.23	0.02
**C**_**area**_	1	5.56*	0.06	0.26	0.01
**N**_**area**_	1	16.10**	4.11*	0.02	3.81
**P**_**area**_	1	0.79	0.10	0.03	3.79

GRM table for step-wise multiple regression of isotope discrimination (Δ^13^C) on leaf mass per area (LMA), C content per unit leaf area (C_area_), N content per unit leaf area (N_area_), P content per unit leaf area (P_area_), stomatal density (SD) and stomatal length (SL) in leaves of four species across two altitudinal gradients on two bedrocks. The table includes degrees of freedom (d.f.) and F values.

Significant values in bold.

* p < 0.05

** p < 0.01

The foliar Δ^13^C was negatively correlated with leaf growth in all species on silicate bedrock but not on carbonate bedrock (S1 Table; Table 6).

**Table 6:** Correlations of C isotope discrimination with leaf growth rates in the four species.

	*V*. *myrtillus*		*V*. *vitis-idaea*		*H*. *alpina*		*C*. *villosa*	
	C	S	C	S	C	S	C	S
**Leaf growth**	- 0.15	- 0.55**	-0.19	- 0.58**	0.00	- 0.65**	- 0.39	- 0.80**

Pearson’s correlation coefficients on carbonate bedrock (C) and silicate bedrock (S). N = 30.

Significant values in bold.

* p < 0.05

** p < 0.01

## Discussion

The results of this study partly supported our first hypothesis. As expected, the foliar Δ^13^C was negatively correlated with LMA and its proxy C_area_ in all four species independent of bedrock type.

The foliar Δ^13^C also presented negative correlations with foliar nutrient contents but such correlation was much stronger for N_area_ than for P_area_. This means that low foliar Δ^13^C always was associated with high-LMA leaves but not necessarily with higher foliar nutrient status. Indeed, the foliar Δ^13^C of all four species was lowest at the uppermost sites (2000–2200 m) where light levels were much higher than in closed forests at lower altitudes. High LMA in high-altitude plants can be due to a direct effect of light because high irradiance enhances leaf thickness through enhanced development of palisade parenchyma [11]. Changes in LMA across altitudinal gradients can also reflect a side effect of cold temperatures through temperature-mediated growth modulation [7]. As LMA did not change gradually across the gradients but exhibited a sudden increase above 2000 m light, more than temperature, appeared to be the primary factor affecting LMA in the four species. As the foliar Δ^13^C reflects the balance between demand and supply of CO_2_, low C isotope discrimination can be caused by reduced g_s_ if high light levels are associated with water stress. The foliar Δ^13^C of the four species was unrelated to soil VWC. As single measurements of soil VWC do not account for seasonal variations in the soil hydric status we cannot rule out the hypothesis that the plants underwent some degree of water limitation at the high-altitude sites above treeline. Although precipitation does not increase with altitude in the study region [20], evapotranspiration always decreases across altitudinal gradients in humid mountains like the Alps [21]. So, we believe that light, much more than soil moisture, was the main driver of changes in the foliar Δ^13^C, as observed in several other studies [22, 23, 24, 25].

Further support to this conclusion could be drawn from the altitudinal patterns of SD and SL. Contrary to our hypothesis, the foliar Δ^13^C was negatively related to SD and unrelated to SL. Furthermore, while SD generally increased with increasing altitude SL did not change across the altitudinal gradients. Should the plants experience water limitation at the high-altitude sites, higher SD would be accompanied by lower SL because high density of small stomata represents an effective adaptation to cope with water stress [26, 27]. If higher SD was accompanied by higher g_s_ this should increase rather than decrease foliar Δ^13^C at high altitude, as foreseen in our first hypothesis. So, some other mechanisms depending on SD probably compensated the potentially stimulating effect of increased g_s_ on foliar Δ^13^C. A possible explanation is that higher SD enhances CO_2_ fixation by photosynthesis more than water loss through transpiration at high altitudes. Bucher et al. [5] observed negative correlations of foliar Δ^13^C with both g_s_ and light-saturated net CO_2_ exchange rates in several species across altitudinal gradients in the Northern Alps. Higher SD may thus enhance net photosynthesis at a faster rate than g_s_ because of indirect effects of atmospheric composition at high altitudes [28, 29]. We found SD to increase across the altitudinal gradients in three out of the four species. This supports the results of previous studies finding SD to increase under high light levels and/or low p_a_ in most species [30, 31]. However, *C*. *villosa* did not exhibit higher SD at high altitudes. In addition, SD influenced C isotope discrimination across the gradients in *V*. *vitis-idaea* and *H*. *alpina*, but not in *V*. *myrtillus* and *C*. *villosa*. This suggests that stomatal control on foliar Δ^13^C differed among species and possibly among PFT as both *V*. *myrtillus* and *C*. *villosa* have deciduous leaf habit whereas *V*. *vitis-idaea* and *H*. *alpina* are evergreen or wintergreen, respectively.

Bedrock geology could influence C isotope discrimination by affecting soil nutrient status [14, 15]. Bedrock geology had poor influence on soil N content but strongly conditioned soil P content (Fig 2). Previous studies pointed to high N_area_, associated with high LMA, as the main cause accounting for lower foliar Δ^13^C consequent to improved photosynthetic capacity in high-altitude plants [32, 33] If bedrock geology played a major role in controlling C isotope discrimination, this would imply positive relationships between foliar Δ^13^C and leaf growth rates because the foliar Δ^13^C integrates photosynthetic activity throughout the period of leaf formation [34]. Indeed, N_area_ has often been related to photosynthetic capacity [35]. However, if the foliar Δ^13^C depended on higher carboxylation efficiency deriving from higher N_area_ this would necessarily imply negative correlations between foliar Δ^13^C and leaf growth rates on both parent materials, that was not the case (Table 6). On the other hand, P_area_ did vary strongly across altitudinal gradients as a consequence of differing soil P content on the two parent materials. Increasing P_area_ across the altitudinal gradient on carbonate bedrock was associated with decreasing leaf growth rate presumably because of stoichiometric imbalance due to low N : P ratios at high-altitude sites [16]. Again, if the foliar Δ^13^C depended on indirect effects of nutrient ratios on growth potential this would imply positive correlations between foliar Δ^13^C and leaf growth rate on carbonate bedrock, that were not found in our study.

In summary, C isotope discrimination across altitudinal gradients were similar on the two parent materials even if the foliar nutrient status varied much across the gradients depending on bedrock geology. Light level shaped anatomical leaf traits, primarily LMA, that most strongly affected foliar Δ^13^C by hampering CO_2_ diffusion in high-LMA leaves. The foliar Δ^13^C was secondarily influenced by SD, whose relative importance in determining C isotope discrimination differed among species and/or PFT. This means that leaf morphology, rather than growth potential depending on foliar nutrient status resulting from varying soil nutrient contents on different bedrock types, was the main factor controlling foliar C isotope discrimination across the altitudinal gradients investigated.

## Supporting information

S1 Table Leaf growth and mass-based nutrient concentrations. Foliar δ^13^C, foliar traits and leaf growth rates across altitudinal gradients on the two bedrock types in the four species.
(XLSX)
